# The Skin-Resident Immune Network

**DOI:** 10.1007/s13671-013-0063-9

**Published:** 2013-11-28

**Authors:** Szun S. Tay, Ben Roediger, Philip L. Tong, Shweta Tikoo, Wolfgang Weninger

**Affiliations:** 1 Centenary Institute, Locked Bag 6, Newtown, NSW 2042 Australia; 2 Discipline of Dermatology, University of Sydney, Sydney, 2006 NSW Australia; 3 Department of Dermatology, Royal Prince Alfred Hospital, Camperdown, 2050 NSW Australia

**Keywords:** Innate immune system, Langerhans cells, γδ T cells, Innate lymphoid cells, Dendritic cells, Macrophages, Mast cells

## Abstract

The skin provides an effective physical and biological barrier against environmental and pathogenic insults whilst ensuring tolerance against commensal microbes. This protection is afforded by the unique anatomy and cellular composition of the skin, particularly the vast network of skin-associated immune cells. These include the long-appreciated tissue-resident macrophages, dendritic cells, and mast cells, as well as the more recently described dermal γδ T cells and innate lymphoid cells. Collectively, these cells orchestrate the defense against a wide range of pathogens and environmental challenges, but also perform a number of homeostatic functions. Here, we review recent developments in our understanding of the various roles that leukocyte subsets play in cutaneous immunobiology, and introduce the newer members of the skin immune system. Implications for human disease are discussed.

## Introduction

**Fig. 1:**
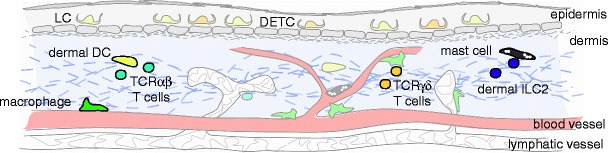
Schematic of skin-resident immune cells. *DETC* dendritic epidermal T cells; *DC* dendritic cells; *ILC2* group 2 innate lymphoid cells; *LC* Langerhans cells

The skin is a unique organ that serves as an interface between the host and the environment, providing a mechanical and biological barrier against chemical, physical and pathogenic insults. Anatomically, the skin comprises two distinct compartments: the epidermis, an avascular layer mainly composed of keratinocytes, and the dermis, a fibroblast-rich network of collagen and elastin fibers that provides the skin with strength and elasticity. The dermis also contains capillary and lymphatic vessels, which serve as the entry and exit portals for immune cells. Additional skin appendages such as hair follicles, sebaceous glands and sweat glands, as well as nerve endings are also found in the dermis [1]. The skin is home to a number of immune populations that reside in both the epidermis and dermis. These cells ensure protection against pathogens whilst maintaining tolerance to innocuous antigens, but also contribute to the pathology of a number of inflammatory skin diseases. This immune network is comprised principally of tissue-resident phagocytes, antigen-presenting cells, mast cells and T lymphocytes, as well as innate lymphoid cells (Fig. 1). Individually, these leukocyte subpopulations perform specialized functions that collectively afford the host its ability to respond to a variety of environmental challenges. In recent years, our understanding of skin immunology has been transformed, with many new insights into both the ontogeny and function of most of the skin-resident immune cells. These developments include the discovery of two hitherto unknown leukocyte populations, the dermal γδ T cells and group 2 innate lymphoid cells. Here, we review the recent advances in our understanding of the functional diversity of the different immune cell subsets and their role in the cutaneous immune response.

## The Skin-Resident Innate Immune Cells

### Macrophages

Macrophages represent a key sentinel population for invading pathogens and tissue damage. These cells also perform developmental and homeostatic functions [2]. Much of our understanding of their roles in vivo has derived from studying macrophage-deficient mice, which include mice lacking the cytokine colony-stimulating factor-1 (CSF-1) or its receptor [3–5]. These animals exhibit a number of developmental and metabolic deficiencies, including defects in iron and erythrocyte homeostasis [6]. Dysregulation or inappropriate activation of macrophages can lead to proinflammatory conditions, and macrophages have been implicated in a number of inflammatory diseases such as atherosclerosis, type 2 diabetes, and cancer [7].

Macrophages comprise a remarkably diverse and heterogeneous population that is found in most tissues, including the skin [8]. During steady-state conditions, macrophages are the most abundant haematopoietic population in the skin [9, 10], which likely underscores their importance in maintaining skin integrity and function. They are also important in wound healing, and their critical role in promoting wound closure and tissue repair has been confirmed using recently developed, inducible macrophage-depleter mice [11, 12, 13•]. Macrophages are equipped with a vast array of genetically encoded cell surface and intracellular molecules called ‘pattern recognition receptors’, which detect both damage-associated and pathogen-associated motifs [14]. Depending upon the nature of the stimulus (e.g., sterile damage or infection), they can produce a variety of proinflammatory cytokines and chemokines that attract specific immune cell subpopulations from the circulation to the site of injury or pathogen invasion [15]. In the later stages of repair, they are able to switch to a growth-promoting [16] and less inflammatory phenotype [17, 18], and begin to actively phagocytose apoptotic cells, including apoptotic neutrophils [19], thereby promoting the resolution of inflammation. This remarkable ability to acquire a spectrum of functional phenotypes depending on stimuli allows macrophages to coordinate a myriad of context-appropriate responses to environmental challenges.

A number of transgenic macrophage reporter mice have proven particularly useful for studying macrophage biology [20], and have enabled direct visualization of macrophage-pathogen interactions in vivo by both conventional and intravital multiphoton microscopy [21]. Similarly, transgenic mice developed for lineage-tracing experiments have also transformed our understanding of macrophage development. Broadly speaking, macrophages may be tissue-resident or may develop from circulating progenitors in the blood. Developmentally, it was long believed that macrophages derived from circulating monocytes [22], and while that likely holds true for macrophages that arise following inflammation, the origin of tissue-resident macrophages differs markedly. Rather, most of the tissue-resident macrophages in the skin, spleen, pancreas, liver, brain and lung are, in fact, established prenatally, and arise from the yolk-sac or fetal-liver progenitors [23, 24•, 25••, 26]. Under some experimental conditions, tissue-resident macrophages appear to be capable of self-renewing without the need for resupply from the blood [27], although the relative long-term contributions of circulating precursors versus self-renewing populations following inflammation is still under debate [28, 29].

Another layer of complexity lies in the pronounced phenotypic and functional diversity of macrophage subsets. Despite their developmental similarities, the function of macrophages in different organs varies. Red pulp macrophages, for example, are responsible for red blood cell clearance [30], while the microglia in the brain are important for neuronal function [31]. Unsurprisingly, there is wide variation in the receptor usage of macrophages from different tissues [32]. Even within the same organ, there appears to be subset specialization within different microanatomical niches. For instance, macrophages in the different zones of the spleen perform different functions. Marginal zone macrophages are important for trapping blood-borne antigens, whereas red pulp macrophages perform scavenger functions [33].

Within the skin, macrophages have long been thought to be a homogeneous group, primarily serving as a first line of defense against potentially invading pathogens. However, it is likely that skin-resident macrophages are more heterogeneous than previously appreciated. Certainly, macrophages within the skin exhibit a diverse distribution anatomically, where they may be associated with blood vessels (perivascular or “adventitial” macrophages), lymphatic vessels, or may reside in the intervascular space [34, 35]. It is conceivable then, that perivascular macrophages might be particularly well-suited for regulating leukocyte extravasation, based on their proximity to blood vessels. Similarly, perivascular macrophages may also play a role in regulating local iron homeostasis [36], while those associated with lymphatic vessels may be more important during lymphangiogenesis [35]. Whether strict partitioning of macrophage functions exists within the skin, or if their multiple functions are achieved by functional plasticity remains unclear. Further studies are required to decipher the precise role of specific cutaneous macrophage subsets in cutaneous homeostasis and disease, including wound healing, infections, and skin tumors.

### Dendritic Cells

Dendritic cells (DCs) represent a subpopulation of antigen-presenting cells that are largely defined by their capacity to present antigens to naïve T cells, for both the generation of immunity against invading pathogens, as well as tolerance to self-antigens and commensal bacteria [37]. With regard to skin DCs, this function requires the capability to migrate via the lymphatics to skin-draining lymph nodes (LNs), where they initiate and shape the downstream adaptive immune response. DC migration to the LNs requires engagement of the chemokine receptor CCR7, expressed by the DC, with one of its two ligands, CCL19 and CCL21, which are in turn expressed by lymphatic endothelial cells [38]. DC migration from the skin may be triggered by a range of noxious and inflammatory stimuli, but also occurs constitutively during steady-state conditions [39, 40]. Skin DCs can be divided into two major populations: Langerhans cells (LC), which reside in the epidermis, and dermal dendritic cells, which reside in the dermis. Although both populations capture and process antigens within the skin and migrate to draining LNs [41, 42], the origins, developmental requirements and behavior of these two populations are quite distinct, as are their functions.

#### Langerhans Cells

Langerhans cells (LC) reside in the basal and suprabasal epidermis, where they form a network between keratinocytes. Intravital experiments have shown that LCs remain sessile during steady-state conditions, but that their dendrites extend between keratinocytes in a repetitive fashion [43–45], which may enable the sampling of antigens [46, 47]. While it is well-accepted that LCs capture antigens and migrate to skin-draining LNs during both steady-state conditions and following inflammation [40], what instruction they provide to naïve T cells is still hotly debated, with evidence that they can both suppress and initiate skin immune responses [48, 49].

Developmentally, LCs have more in common with macrophages than dermal dendritic cells. LCs develop from a primitive macrophage population during embryogenesis, which can be yolk-sac or fetal liver-derived [25••], and in the absence of inflammation maintain their numbers within the epidermis throughout life by in situ proliferation [41, 50, 51]. This capacity for local self-renewal renders LCs radio-resistant, and the vast majority of LCs do not get replaced by donor bone marrow-derived progenitors following irradiation and stem cell transplantation, a finding that is true of both mice and humans [52, 53, 54•]. LC development requires IL-34 signaling through the CSF-1 receptor [55••, 56••], and LC homeostasis is maintained by TGF-β [57], which is produced by both keratinocytes (paracrine) and LCs themselves (autocrine). During steady-state conditions, a small percentage of LCs constitutively emigrates to the skin-draining LNs [40], but this loss is readily maintained by local proliferation of the remaining LC pool [58]. However, following relatively severe inflammation, for example that induced by ultraviolet irradiation or herpes simplex virus infection, increased numbers of LCs migrate to the LNs [41, 51, 59] (estimated to be approximately 10–20 % of the total pool; B. Roediger unpublished observations). More significantly, if the epidermal niche becomes sufficiently perturbed, LC homeostasis is compromised and requires replenishment from bone marrow-derived monocytes. These monocytes enter the skin in response to inflammatory chemokines, and follow a chemotactic gradient from the base of the hair follicle to the upper epidermis where they differentiate into LCs [60]. It is currently unclear whether there are functional differences between fetal-origin LCs compared to these monocyte-derived LCs. Although the original study suggested that monocyte-derived LCs could proliferate and maintain themselves in situ [51], a recent study suggests that, following the resolution of inflammation, these cells are ultimately out-competed by those of fetal-origin [61].

Despite being discovered over 100 years ago, the exact immunological role of LCs remains controversial [48, 49]. Early studies suggested that LCs were potent stimulators of T cells, but this was based largely on in vitro experiments, and has been challenged by a number of in vivo studies. The identification of Langerin as a LC-specific marker [62] led to models for specific LC depletion [63–65]. However, these models provided inconsistent data regarding their role in adaptive immune responses, with positive, negative and redundant contributions of LCs to contact hypersensitivity responses being reported [66]. The use of langerin-eGFP reporter mice led directly to the discovery of Langerin^+^ dermal dendritic cells, which further confounded interpretations of the LC-depletion models [63–65]. More recently, we and others have exploited the radio-resistance of LCs to exclude the confounding contributions of dermal dendritic cells to naïve T cell responses [39, 67]. Remarkably, LCs were committed to initiating the tolerance of naïve T cells, regardless of the inflammatory stimulus [67]. An immunosuppressive role for LCs in vivo has also been described in other models [68–71], underscoring the difference between the stimulatory behavior of LC in vitro and their suppressive capabilities in vivo. Presumably due to this discrepancy, the murine results have not been confirmed in human studies, which are performed mostly in vitro [72–75].

#### Dermal Dendritic Cells

Dermal dendritic cells (DDCs), in contrast to LCs, are a more heterogenous population that relies upon continuous resupply from bone marrow-derived progenitors. In both mice and men, the dermis contains multiple DDC subsets, although the functional specialization of each subset remains largely unknown. In mice, the majority of DDCs express CD11b but not CD103 (CD11b^hi^CD103^-^), but there are also CD11b^lo^CD103^+^Langerin^+^ and CD11b^-^CD103^-^ subsets [63–65, 76, 77]. In contrast to the sessile LCs, DDCs are highly mobile and continuously migrate throughout the dermis, presumably as part of their immune-surveillance role [44]. During inflammation, DDCs are mobilized rapidly from the skin and arrive in draining LNs within 48 hours, preceding the arrival of LCs, which peak at day 4 [43, 77]. Thus, DDCs are likely to be responsible for shaping the initial T cell response to skin pathogens.

In addition to their potential to initiate pathogen-specific immune responses [44, 59, 78], skin DDCs are also detectable within cutaneous LNs during steady-state conditions, suggesting that they are important for maintaining tolerance to skin antigens [39]. They have also been implicated in regulation of the immune response to skin damage [79]. It appears that functional specialization amongst the different DDC subsets enables them to fulfill a variety of diverse requirements. The best example is that of CD11b^lo^CD103^+^Langerin^+^ DDCs, which are highly efficient at cross-presenting antigens to naïve CD8^+^ T cells compared to the other DDC subsets [76, 80], and likely promote Th1-type immune responses [78]. In contrast, a CD301b^+^ DDC, distinct from Langerin^+^ DDCs, was recently shown to be important for the generation of Th2 responses [81, 82]. Of note, equivalent subpopulations have been described in human skin, suggesting that many of these functions may be conserved between mice and humans [75, 83]. Indeed, a CD141^hi^ DC population has recently been identified in the dermis that shows both transcriptional and functional equivalence to CD103^+^ DDC [84••], which may have implications for future vaccine design.

### Mast Cells

Mast cells are especially abundant at host-environmental interfaces, including the skin, where they are found in close proximity to the blood vessels in the dermis. Skin mast cells are best known as critical effectors of Th2 immune responses, including allergic inflammatory diseases, in which environmental allergens trigger their release of pre-formed inflammatory molecules such as histamine, a potent vasodilator [85]. This process is generally mediated via the cross-linking of high-affinity IgE-receptors (FcεRI) on the mast cell surface by IgE-bound allergens, which in turn promotes tissue inflammation such as urticaria and angioedema. In addition to their role as Th2 effectors, it has been proposed that mast cells participate in pathogen defense, contact hypersensitivity responses, and wound healing, during which they perform both pro- and anti-inflammatory functions [86]. Indeed, the dual pro- and anti-inflammatory properties of mast cells have confounded our understanding of their role in numerous inflammatory conditions, including allergic diseases such as atopic dermatitis. More controversially, mast cells have been implicated in the pathogenesis of a number of autoimmune disease models [87].

Developmentally, mast cells require signaling from the stem cell factor through the Kit receptor for their survival and development, such that mice with defects in Kit signaling and/or expression also lack mast cells. This deficiency can be restored following intradermal injection of in vitro*-*generated mast cells, which has formed the basis for elucidation of mast cell function in vivo [86, 88]. Studies using these ‘knock-in’ models have implicated mast cells in protection against bacteria, parasites, and viruses in the skin [89–91].

Nevertheless, studies using *Kit*-deficient mice must be interpreted in the context of the additional cellular defects exhibited by these animals, since the Kit signaling pathway is also important for hematopoietic stem and progenitor cell, red blood cell and neutrophil development [92–94]. To overcome these shortcomings, mast cell researchers have more recently developed *Kit*-independent mast cell depleter models, which are currently being used to re-address the role of mast cells in different settings. To date, these studies have confirmed the requirement for mast cells in both the sensitization and effector phases of cutaneous hypersensitivity responses [95] and allergic inflammation [96•], but have questioned their role in autoimmunity [95, 97]. These mice have also been used to demonstrate the contribution of mast cells to pathology in a murine model of atopic dermatitis [98•], which was consistent with the efficacy of anti-IgE therapy in treating patients with severe atopic dermatitis [99, 100].

Mast cells can also be studied by multiphoton microscopy, and it was recently shown that skin mast cells extended cellular processes across vessel walls in vivo in order to acquire IgE from the circulation [101]. We have also used multiphoton microscopy to visualize skin-resident mast cells in situ, where we observed them interacting with group 2 innate lymphoid cells in vivo ([102••]; discussed below).

### γδ T Cell Receptor-Expressing Cells (γδ T cells)

#### Dendritic Epidermal T Cells

In addition to being the home of LCs, the murine epidermis is also home to a population of T cells that have been termed dendritic epidermal T cells (DETC), based on their location and morphology [103]. DETCs are γδ T cells that express the canonical Vγ5/Vδ1 T cell receptor (nomenclature: [104]). They have no human equivalents but are included in this review to provide context to the recently characterized population of dermal γδ T cells that can be found in both mice and humans.

DETCs form tight associations with E-cadherin expressed on keratinocytes [105], and this contributes to their dendritic morphology. Similar to LC, they remain largely immobile, as observed by intravital microscopy [106•]. The ligand for the DETC TCR remains unknown but appears to be constitutively expressed by keratinocytes [107]. DETCs require both IL-7 and IL-15 for their maintenance in the skin [106•, 108–110], where they appear to be important for epidermal homeostasis and repair [111]. However, much of our understanding of DETC function, particularly in vivo, has derived from the use of TCRδ^−/−^ mice, which has been predicated upon the assumption that these cells represented the sole γδ T cell population within the skin. The recent discovery of an additional population of skin-resident γδ T cells, namely the dermal γδ T cells, necessitates a revision of these studies. For instance, while the increased keratinocyte apoptosis and delayed wound healing observed in TCRδ^−/−^ mice is consistent with the role for DETCs in the epidermis [111–114], the impaired leukocyte recruitment during skin infection with *Staphylococcus aureus* may be attributed to functions shared by dermal γδ T cells, in particular IL-17 production [115].

#### Dermal γδ T Cells

Despite the long-held view that DETCs were the sole γδ T cell population within the mouse skin, previous studies had indicated the presence of an additional skin-resident population of γδ T cells that do not express Vγ5 [116, 117]. In transgenic mice engineered to overexpress IL-7 from keratinocytes, spontaneously occurring skin lesions were found to comprise predominantly Vγ5^−^ γδ T cells, both within the dermis and epidermis, with only a few DETC. These γδ T cells could be elicited from IL-7-stimulated skin organ culture, suggesting they were normally skin-resident. More recently, we and others have extensively characterized this dermal γδ T cell subset [106•, 118, 119], confirming they are indeed a dermal-resident population that is both phenotypically and functionally distinct from DETCs.

Dermal γδ T cells constitute 50 % of the total dermal T cell population in mice, 30–50 % of which express the Vγ4 TCR. They are round or amoeboid in morphology, and a significant proportion are migratory [106•], albeit with slower kinetics than TCRαβ T cells [120]. Unlike DETCs, dermal γδ T cells require IL-7 but not IL-15 for their development, and form a long-lived population in the skin that is capable of self-renewal [106•, 121]. Dermal γδ T cells constitutively express IL-23 receptor, CCR6, and RORγt molecules associated with Th17 cells [122]. Indeed, they are able to produce IL-17A in response to stimulation by IL-1β and IL-23 or selected toll-like receptor agonists [106•, 118, 123]. Thus, dermal γδ T cells are likely to be involved in innate pathogen defense by augmenting neutrophil recruitment via IL-17. Recent studies have also revealed the importance of dermal γδ T cells in imiquimod- and IL-23-induced psoriasiform lesions in mice, in which they were the major source of IL-17. Interestingly, it was dermal γδ T cells, not αβ T cells or DETCs, that were important for lesion development [118, 121, 124•]. These findings suggest the intriguing possibility that dermal γδ T cells may contribute to the pathology of human psoriasis, given the pivotal roles of IL-17 and IL-23 in this disease [125–127], although this remains speculative.

Human skin also contains a population of dermal γδ T cells, most of which express Vδ1 TCR, contrasting with the γδ T cells found in human peripheral blood that comprise largely Vδ2^+^ cells. Whether this population is the human equivalent to the murine dermal γδ T cell remains unclear, although the evidence to date suggests that the two populations do not equate. In contrast to murine γδ T cells, which are pre-committed to IL-17 production in the embryonic thymus [121, 128], human dermal Vδ1^+^ T cell lines produce TNFα and IFNγ when stimulated in culture [129, 130]. Human blood contains a Vγ9^+^Vδ2^+^ subset expressing cutaneous lymphocyte-associated antigen (CLA), the skin-homing receptor. Importantly, these circulating Vγ9^+^Vδ2^+^ cells produced IL-17 after bacterial infection and have been identified in psoriatic lesions [131•]. Understanding IL-17-producing innate cells and their functional and survival requirements could lead to targeted therapies, for instance, RORγt antagonists.

### Innate Lymphoid Cells

Innate lymphoid cells (ILCs) are a family of newly described cells derived from a common lymphoid progenitor, and are identified by their lack of lineage marker expression (T-cell receptor, B-cell receptor, myeloid and/or DC markers) and their lymphoid morphology [132]. They are further subcategorized according to their developmental requirements and the cytokines that they produce: group 1 ILCs (ILC1) are T-bet dependent and produce IFNγ; group 2 ILCs (ILC2) are GATA-3 dependent and produce the type 2 cytokines IL-5 and IL-13; and group 3 ILCs (ILC3) are RORγt-dependent and produce IL-17, IL-22, or both (nomenclature: [133]).

Although originally described in mucosal tissues, significant numbers of ILC2s have been identified in the dermis of both mice and humans [102••, 134••]. Specifically, they were one-third as numerous as T cells and comprised 5–10 % of CD45^+^ cells isolated from murine skin. Like other ILCs, dermal ILC2s required IL-7 for their development and survival, and could be replenished by bone marrow-derived cells following irradiation. Dermal ILC2s constitutively produced IL-13, and could upregulate IL-5 and IL-13 production when activated by systemic IL-2 treatment [102••], or by topical administration of the vitamin D analogue calcipotriol [134••]. Intravital imaging of dermal ILC2s revealed that these cells scan the dermis but also frequently stop to interact with skin-resident mast cells, another effector of type 2 responses [102••]. Interestingly, ILC2s were enriched in human atopic dermatitis lesions [134••], implicating this population in the pathology of eczema.

Although there is no evidence yet to date that ILC1s or ILC2s reside within normal skin, ILC3s were recruited to imiquimod-induced psoriasiform lesions [124•]. Whether equivalent populations of ILC3s are enriched in human psoriasis remains to be determined.

## Concluding Remarks

Research over the past few years has shed new light on the complex functions of skin-resident immune cells in homeostasis and inflammation. The discovery of novel cell populations, such as dermal γδ T cells and ILC2s, has expanded our knowledge of innate immune sensing and early responses towards pathogens entering the skin. How exactly epidermal and dermal inhabitants interact with each other and the environment and coordinate downstream adaptive immunity is still largely unexplored. The development of transgenic reporter mice with fluorescently tagged immune cell subsets in combination with advanced imaging approaches provides a unique opportunity for furthering our understanding of cutaneous biology in the steady-state and during disease conditions in the years to come.
